# An Abnormal Presentation of a Popliteal Artery Mass and a Unique Cause of Claudication

**DOI:** 10.1155/cro/9960715

**Published:** 2026-04-21

**Authors:** Jamil Haddad, Majed Alzahabi, Saif L. Juma, Nicholas David Cominos, Shariff K. Bishai

**Affiliations:** ^1^ Department of Orthopedic Surgery, McLaren Macomb, Mount Clemens, Michigan, USA; ^2^ Michigan State University College of Osteopathic Medicine, East Lansing, Michigan, USA, msu.edu; ^3^ Wayne State University School of Medicine, Detroit, Michigan, USA, wayne.edu; ^4^ Department of Orthopedic Surgery, Henry Ford Macomb, Clinton Township, Michigan, USA; ^5^ Department of Orthopaedic Surgery, Detroit Orthopaedic Institute, Troy, Michigan, USA

## Abstract

Multiple hereditary exostosis (MHE) is a rare autosomal‐dominant disorder marked by multiple osteochondromas arising from the metaphyses of long bones. Parameniscal cysts, often linked to meniscal tears, can also expand and compress nearby vessels, posing a potential vascular risk. We present a 28‐year‐old male with MHE and a parameniscal cyst causing popliteal artery compression. The patient’s intermittent claudication resolved completely following surgical cyst decompression and meniscal repair. This case report highlights the importance of considering vascular compression in patients with MHE who present with lower extremity symptoms.

Key Clinical Message

Popliteal artery compression can occur secondary to parameniscal and adventitial cyst formation in patients without traditional vascular risk factors. Awareness of atypical vascular etiologies of claudication is essential, particularly in patients with underlying musculoskeletal pathology, to ensure timely diagnosis and appropriate multidisciplinary management.

## 1. Introduction

Multiple hereditary exostosis (MHE) is a rare inherited disorder with an autosomal‐dominant pattern, marked by the development of cartilaginous bone tumors at the metaphyses of bones that grow through endochondral ossification [1]. Although uncommon, these osteochondromas may compress nearby neurovascular structures, potentially causing symptoms such as numbness, blood vessel blockages, dissections, and aneurysms [2].

Parameniscal cysts are fluid‐filled sacs with a prevalence of approximately 4%, more frequently affecting males [3]. These cysts typically develop in the posterior region of the knee, often situated between the semimembranosus tendon and the medial head of the gastrocnemius muscle [4]. They commonly arise from underlying knee conditions, such as osteoarthritis, rheumatoid arthritis, or meniscal tears, which lead to excessive synovial fluid production [5, 6]. Trauma, degenerative changes, and autoinflammatory processes contribute to their formation, with most cases (98%) linked to horizontal meniscal tears. The widely accepted mechanism suggests that synovial fluid extrudes through the tear, facilitating cyst development [7, 8].

In certain cases, a parameniscal cyst can progress into an adventitial cyst, leading to compression of nearby structures. The popliteal artery is typically affected, resulting in symptoms of claudication in the lower extremity [9]. While physical exams may appear normal, occasionally a popliteal bruit or reduced distal pulses during knee flexion can be noted. Diagnosis is often confirmed through imaging techniques such as ultrasound, CT scans, MRI, or angiography [9].

The popliteal artery extends from the superficial femoral artery as it travels through the adductor magnus hiatus. In the popliteal fossa, it runs adjacent to the popliteal vein and ultimately branches into the anterior tibial artery and the tibioperoneal trunk at the lower edge of the popliteus muscle, aligned with the tibial tuberosity [10]. Compression of this artery by an adventitial cyst can result in popliteal artery compression syndrome, potentially presenting with symptoms such as claudication, swelling, or ischemic signs in the affected limb [9].

Treatment for symptomatic parameniscal cysts depends on the presence of a concurrent meniscal tear. Common approaches include arthroscopic partial meniscectomy or meniscus repair, often combined with cyst decompression. In cases where the cyst is extensive or recurrent, open cyst decompression along with meniscus repair may be necessary to fully relieve symptoms and restore joint function [11].

In this report, we present the importance of considering atypical vascular causes in patients with intermittent lower extremity symptoms. Vascular complications in MHE are particularly rare, with only a small number of cases involving lower extremity arteries in young individuals.

## 2. Case Description

In August 2021, a 28‐year‐old male with a history of MHE and a resolved left medial meniscus tear, previously treated with partial medial meniscectomy, presented with complaints of left calf claudication and numbness occurring after ambulating 200 m or more. These symptoms consistently resolved with rest and had been present for 6 months, progressively worsening over the past 4 weeks. The patient also reported a similar, less severe episode 3 years earlier, triggered exclusively by weightlifting, which resolved spontaneously within 3 months. At that time, a diagnostic workup, including X‐rays and Stryker needle testing, ruled out exertional compartment syndrome.

On physical examination, the patient was an athletic young male in sinus rhythm, with a heart rate of 60 beats/min and a blood pressure of 118/70 mmHg. Dorsalis pedis and posterior tibial pulses were palpable on the left lower extremity but were reduced in strength compared to the contralateral side. Doppler ultrasound of the left lower extremity revealed monophasic pulses and an ankle–brachial index (ABI) of 0.76, in contrast to triphasic pulses and a normal ABI of > 1.1 on the right. The skin showed no rashes or signs of trauma. A palpable mass was noted within the popliteal fossa. The knee was without effusion, demonstrated full range of motion, was stable to stress testing, and had a negative McMurray test.

**Figure 1:**
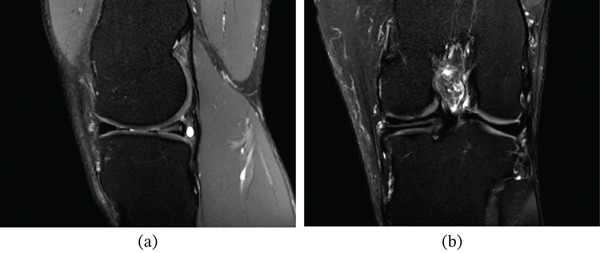
Sagittal (a) and coronal (b) T2‐weighted MRI images demonstrating a horizontal cleavage tear through the posterior horn of the medial meniscus.

**Figure 2:**
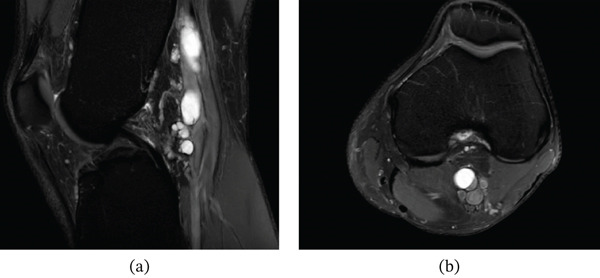
Sagittal (a) and axial (b) T2‐weighted MRI images of the knee revealing a multiloculated, homogeneous cystic lesion extending proximally adjacent to the popliteal artery, causing compression of the surrounding structures.

An MRI from August 2021, with and without contrast, showed a horizontal cleavage tear through the posterior horn of the medial meniscus (Figure 1). Additionally, a tubular, multiloculated cystic lesion was identified, extending directly from the meniscal tear into the popliteal fossa and compressing the neurovascular bundle (Figure 2). The lesion measured up to 7.9 × 1.4 × 1.5 cm and did not exhibit contrast enhancement.

The patient was scheduled for a left knee arthroscopy followed by open cyst removal 2 weeks later to allow for postsurgical swelling to subside. During the arthroscopy, the meniscal cleavage tear was addressed, and the meniscus was repaired. Subsequently, during the open cyst excision, the cyst was identified as an adventitial popliteal artery cyst. The cyst stalk, which extended into the knee joint from the meniscus, was isolated and ligated. After evacuating the jelly‐like cystic material, the arterial tunica media was inspected and found to be intact. The artery returned to its normal caliber once the compressive pressure was relieved. The excess adventitial tissue was debrided, and the artery was repaired. Postprocedure Doppler examination confirmed triphasic flow and restoration of normal blood flow to the most distal aspect of the left lower extremity.

Follow‐up evaluation revealed an ABI of 1.10 on the left and 1.13 on the right, with complete resolution of claudication symptoms in the left lower extremity. As of October 2024, the patient continues to do well, with no recurrence of claudication.

## 3. Discussion

MHE is a rare inherited skeletal disorder characterized by the development of cartilage‐capped bony projections, commonly found near the ends of long bones [12]. Although osteochondromas associated with MHE are generally benign, complications can arise in approximately 4% of cases, leading to issues such as bone fractures, skeletal deformities, nerve compression, and vascular involvement [13].

Vascular complications, while rare, pose significant risks, particularly in patients with MHE or isolated osteochondromas. Among these, the popliteal artery, located behind the knee, is the most frequently affected due to its proximity to common sites of osteochondromas, such as the distal femur and proximal tibia [14]. The limited mobility of the popliteal artery makes it especially susceptible to mechanical pressure exerted by these bony growths during knee movement, potentially causing damage to the arterial wall and the formation of pseudoaneurysms [15]. These arterial changes can compromise blood flow, resulting in symptoms such as leg pain, claudication, or weakness [13].

Parameniscal cysts often develop from untreated or undiagnosed meniscal tears or degenerative knee conditions [4]. While most parameniscal cysts remain small and asymptomatic, this case highlights a more severe progression where the cyst developed into an adventitial cyst, resulting in popliteal artery compression syndrome [4, 11]. Research suggests that multiple local factors may contribute to cyst formation, including prior trauma, inflammatory processes, and underlying joint pathology. However, the prevailing theory is that a meniscal tear allows synovial fluid to escape, which accumulates in the surrounding parameniscal soft tissue, ultimately leading to cyst development [8, 11, 16]. MRI is essential for accurately diagnosing such lesions and guiding subsequent treatment [17].

A parameniscal cyst may enlarge and compress adjacent vascular structures, and in rare cases, this process can extend to involve the adventitial layer of the popliteal artery, resulting in popliteal artery compression syndrome [18]. Cystic adventitial disease (CAD) was first described by Atkins and Key in 1947 and is an uncommon nonatherosclerotic vascular condition that accounts for a very small proportion of vascular disorders [9, 19]. It most often affects young to middle‐aged, otherwise healthy patients, with a higher prevalence among males [9, 19]. The cystic lesions contain a gelatinous mucin‐rich substance composed primarily of proteoglycans and mucopolysaccharides that accumulates within the adventitial layer and produces external narrowing of the arterial lumen [9]. This narrowing leads to impaired blood flow and ischemic symptoms in the affected extremity [9, 20]. Although the popliteal artery is involved in most cases, less frequent involvement of other arterial segments has been reported, including the external iliac, femoral, radial, ulnar, brachial, and axillary arteries [9, 20–22].

Adventitial cystic disease presents most commonly in middle‐aged men but has been reported across a broad age range, often manifesting as exertional calf claudication in otherwise healthy individuals [23]. Management is guided by symptom severity and the condition of the affected vessel, with both operative and nonoperative strategies described [20, 23]. Surgical management is typically favored due to its low recurrence rate. Surgical techniques include interposition grafting of the affected vessel segment, which provides the highest long‐term patency and was utilized in this case, as well as cyst excision and complete resection of the affected adventitia [9].

In addition to open surgical techniques, minimally invasive strategies have been described for both parameniscal and adventitial cysts. Ultrasound‐guided percutaneous aspiration of cystic lesions has demonstrated successful arterial decompression and symptom resolution in select cases, particularly when the arterial wall remains structurally intact and there is limited cyst extension [24]. Image‐guided aspiration may offer a less invasive alternative with a shorter recovery time; however, recurrence rates are variable, especially when the underlying meniscal tear or intra‐articular communication is not addressed. For parameniscal cysts specifically, arthroscopic management of the associated meniscal tear combined with cyst decompression is often recommended to reduce recurrence risk.

Rosiak et al. [24] demonstrated two cases of CAD involving the popliteal artery, where calf claudication was resolved through percutaneous ultrasound‐guided cyst aspiration.. Both cases demonstrated successful arterial decompression, reduced cyst size, and restored blood flow. The patients experienced complete symptom resolution and were free of calf pain 5 years postprocedure [24]. Conservative management of CAD involves monitoring for potential spontaneous resolution, though cysts typically enlarge and worsen stenosis over time [9]. Jibiki et al. [25] reported a case of a 44‐year‐old man with CAD of the popliteal artery who developed rapid‐onset claudication that improved unexpectedly within 5 weeks, with imaging revealing near‐complete cyst resolution. This highlights the rare possibility of spontaneous regression, supporting conservative management as a viable option in some cases.

In this case, the patient, who had a history of MHE, suffered a traumatic injury that resulted in a horizontal cleavage tear of the medial meniscus. Initial treatment involved a partial medial meniscectomy to alleviate symptoms while leaving the tear partially intact. Properly addressing both the cyst and the meniscal tear is critical to achieving the best possible outcome for the patient. To optimize surgical conditions, the meniscal repair was performed 2 weeks before the open posterior cyst removal, allowing time for knee swelling to subside. Additionally, a vascular surgery team was present during the cyst excision to manage the artery as necessary, depending on intraoperative findings during cyst decompression.

## 4. Conclusion

This case demonstrates the importance of considering rare causes of claudication in young, athletic individuals. The patient’s history of MHE complicated by a medial meniscus tear contributed to the development of a parameniscal cyst that progressed to an adventitial cyst, resulting in popliteal artery compression. Understanding the potential for vascular involvement in MHE is essential for early diagnosis and management, as timely surgical intervention can prevent severe complications and improve patient outcomes. The successful treatment involved a staged surgical approach of initial meniscus repair followed by open cyst excision. Postoperative outcomes demonstrated full resolution of symptoms and restoration of normal arterial flow, emphasizing the effectiveness of surgical intervention for adventitial cystic disease in achieving favorable long‐term results.

## Author Contributions

N.D.C. and S.L.J. contributed to manuscript drafting and literature review. M.A. contributed to data collection and manuscript preparation. J.H. contributed to surgical management and conceptualization of the report. S.K.B. provided senior supervision and critically revised the manuscript.

## Funding

No funding was received for this manuscript.

## Disclosure

All authors reviewed and approved the final manuscript.

## Ethics Statement

The authors have nothing to report.

## Consent

Written informed consent was obtained from the patient for publication of this case report and any accompanying images.

## Conflicts of Interest

The authors declare no conflicts of interest.

## Data Availability

The data that support the findings of this study are available from the corresponding author upon reasonable request.
